# Epidemiology and Outcomes of Patients Presenting to United States Emergency Departments with Vaginal Bleeding

**DOI:** 10.5811/westjem.49015

**Published:** 2026-02-03

**Authors:** Jake Mooney, Emily Shearer, Shay Strauss, Chuyun Xu, Janette Baird, Siraj Amanullah

**Affiliations:** * Alpert School of Medicine at Brown University, Department of Emergency Medicine, Providence, Rhode Island; † Brown School of Public Health, Providence, Rhode Island; ‡ Central Oregon Emergency Physicians, Bend, Oregon

## Abstract

**Introduction:**

There are significant gaps in knowledge regarding the epidemiology, management, and outcomes of patients presenting to the emergency department (ED) with vaginal bleeding.

**Methods:**

This was a retrospective, successional cross-sectional study using data from the National Hospital Ambulatory Medical Care Survey (NHAMCS) examining all adult patients presenting to EDs with vaginal bleeding from 2011–2019. Patients were stratified by age, race/ethnicity, and pregnancy status. Main outcomes were ultimate outcome severity, presenting vital signs, and diagnostic tests performed. We defined high-severity outcome as any patient who was dead on arrival, died in the ED, or during that hospitalization; any patient admitted to the intensive care or stepdown units or to the cardiac catheterization lab or the operating room; or patients transferred to a non-psychiatric hospital. Moderate severity was defined as any patient admitted to floor-level care, held in observation, or transferred to a psychiatric hospital. We defined low-severity outcome as any patient discharged home.

**Results:**

Patients presenting with a chief complaint of vaginal bleeding comprised 1.3% (95% CI, 1.2–1.4%,) of all ED visits, representing 14,620,933 total encounters. Of these patients, 53.0% (95% CI, 49.4–56.7%) were identified as pregnant. There was a lower prevalence of White patients presenting with this complaint compared to White patients presenting with any chief complaint (45.6% [95% CI, 41.9–49.4] vs 60.3% [95% CI, 57.7–62.8%]), with a reciprocal higher prevalence of Hispanic patients (21.1% [95% CI,17.7–24.5%] vs 13.2% [95% CI, 11.7–14.8%]). The majority of patients (88.1%, 95% CI, 86.1–90%) were classified as having a low-severity outcome, 10.3% (95% CI, 8.5–12.1%) were classified as moderate-severity, and 1.6% (95% CI,1.0–2.2%) as high-severity. Patients who were ultimately classified with high-severity outcomes had significantly higher shock indices at presentation and shorter wait times than patients with low-severity outcomes (0.75 [95% CI, 0.72–0.78] vs 0.68 [95% CI, 0.67–0.69], and 23.4 minutes [95% CI, 17.1–29.8] vs 41.7 minutes [95% CI, 37.1–46.4], respectively), despite no difference in median Emergency Severity Index triage score (2.5 [IQR 2.1–2.8] v 2.6 [IQR 2.2–2.9]). A quarter of patients (24.3% [95% CI, 20.8–27.7%]) received a pelvic exam: there were no significant differences in pelvic exam rate by age, pregnancy status, race/ethnicity, or ultimate outcome severity.

**Conclusion:**

Although most patients presenting to EDs with vaginal bleeding are discharged home, current triage models do not appear to appropriately risk-stratify higher risk patients. Disparities in presentation exist.

## INTRODUCTION

Vaginal bleeding is a common presenting chief complaint in the emergency department (ED), sometimes requiring stabilization or resuscitation. It has been estimated that 5% of ED visits are for a chief complaint of vaginal bleeding;^1^ however, the true rate is unknown. Some studies have quantified the number of patients presenting with chief complaints related to vaginal bleeding; however, these studies have focused on subsets of this chief complaint (eg, vaginal bleeding in early pregnancy or abnormal uterine bleeding in specific age groups).^2^–^6^ These studies do not consider all-comers presenting with vaginal bleeding but rather stratify this group based on clinical information not always available at time of triage (eg, the results of a pregnancy test). This distinction is important because if emergency physicians do not have this information at time of initial presentation, they cannot rely on these distinctions for initial triaging, risk-stratification, or workflow.

In addition, apart from one study examining characteristics associated with inpatient hospitalization in the abnormal uterine bleeding population,^2^ no study has examined characteristics associated with severe outcomes for patients who presented tn the ED for vaginal bleeding. Today, with increasing wait times and increased reliance on effective triage models, it is necessary to examine this group as a whole and identify factors associated with poor outcomes prior to testing. Moreover, as restrictions on safe abortion care increase across the United States (US),^7^ more patients with concerns about vaginal bleeding may turn to EDs as a place of care. Understanding the epidemiology of this common chief complaint, as well as who is at risk for severe outcomes, is more important than ever.

Our primary objective in this study was to describe the demographics of patients presenting to US EDs with a chief complaint related to vaginal bleeding. The secondary objective was to further stratify this group by pregnancy status, age, race/ethnicity, and outcome severity. The tertiary objective was to evaluate initial Emergency Severity Index (ESI) score, initial Shock Index (SI), use of specific diagnostic tests (computed tomography [CT], ultrasound, and pelvic exam), and final disposition, by pregnancy status, age, race/ethnicity, and outcome severity.

## METHODS

### Study Design

This was a retrospective successional cross-sectional study using the National Hospital Ambulatory Medical Care Survey (NHAMCS) from the years 2011–2019. The NHAMCS uses a cross-sectional, three-stage probability sampling design and is based on a national sample of visits to EDs in non-institutional general and short-stay hospitals, exclusive of federal, military and Veterans Administration hospitals. The NHAMCS has been conducted annually since 1992.^8^ We purposefully excluded the year 2020 in our study due to known changes in ED volume during the COVID-19 pandemic, which are not thought to be due to true changes in patient need.^9^,^10^ Patients and/or the public were not involved in the design of this study.

As a retrospective observational study, we adhered to the joint statement on Strengthening the Reporting of Observational Studies in Epidemiology (STROBE statement), including the STROBE checklist for method reporting.^11^ This study was deemed exempt by our institution’s institutional review board.

**Population Health Research Capsule**

What do we already know about this issue?

*Vaginal bleeding is a common presenting chief complaint to United States emergency departments (EDs)*.

What was the research question?

*What are the epidemiology and outcomes of patients presenting to the ED with vaginal bleeding, and what factors are associated with high-severity outcomes?*

What was the major finding of the study?

*Vaginal bleeding comprised 1.3% (95% CI, 1.2–1.4%,) of all ED visits; 1.6% (95% CI, 1.0–2.2%,) had a high-severity outcome*.

How does this improve population health?

*This is the first study to examine the incidence, demographics, and outcomes of all adult patients presenting to United States EDs with vaginal bleeding*.

### Study Participants

Patients ≥18 years of age presenting to the ED with a chief complaint of vaginal bleeding were included. Specifically, this study included the population of patients experiencing an increase in frequency or quantity of vaginal bleeding. Rather than using retrospective *International Classification of Diseases*, Revisions 9 and 10, (*ICD*) diagnostic codes,we used the presenting complaint, which is coded in the NHAMCS dataset, to identify inclusion criteria. This enabled us to examine clinically practical implications for triage in the emergency setting. (Included NHAMCS codes are available in Appendix I.)

Not all patients who present with a chief complaint related to vaginal bleeding may identify as female. The NHAMCS dataset does not allow for a selection of patient sex other than male or female. No patient listed as “male” in the dataset met inclusion criteria described above; regardless, we used the non-binary term “patient” in this paper.

### Term and Variable Definitions

#### Age Stratification

Due to the clinical importance of menopausal status in evaluating vaginal bleeding, we stratified patients by age based on guidelines from the American College of Obstetricians and Gynecologists (ACOG): pre-menopause (18–44); peri-menopause (ages 45–55); and post-menopause (≥ 56 years of age).^12^

#### Race/Ethnicity

Race/ethnicity was determined using NHAMCS categories: White (non-Hispanic); Black (non-Hispanic); Hispanic; and other (non-Hispanic).

#### Pregnancy Status

Pregnancy status is not documented in the NHAMCS data, and no data on pregnancy testing at the time of ED visit was available. Therefore, patients were identified as pregnant if either any of their reasons for the visit were specifically related to pregnancy, or if any of their retrospective *ICD* diagnostic codes were related to pregnancy, childbirth, or the puerperium. The transition from *ICD-9* to *ICD-10* in 2015 was considered and accounted for. See Appendix I for inclusion codes.

#### Outcome Severity Stratification

We further stratified patients by their outcomes to identify their risk status on presentation to the ED. Patients with high-severity outcomes were those who met any of the following criteria: dead on arrival; died in the ED; died during that hospitalization; admitted to the intensive care or stepdown units; admitted directly to the cardiac catheterization lab or the operating room; or transferred to a non-psychiatric hospital. Patients with moderate-severity outcomes were admitted to a floor level of care or mental health/detox unit, were held in an observation unit (regardless of whether they were then admitted or discharged), or were transferred to a psychiatric hospital. Patients with low-severity outcomes were those discharged home directly from the ED.

#### Shock Index

The Shock Index is the heart rate divided by the systolic blood pressure. This is a validated metric for increased mortality^13^ and the need for blood products, fluids and vasopressors,^14^,^15^ as well as increased incidence of peri-intubation cardiac arrest.^16^ A SI ≥ 0.9 is generally considered abnormal and is used as the cutoff in the literature.

#### Pelvic Exam

We determined whether a patient received a documented pelvic exam using NHAMCS data, which contains a field indicating whether this test was performed by any clinician at any point during the patient’s ED encounter.

### Data Analysis

All eligible patient encounters from the study years were included for analysis. We reported demographics as proportions and stratified them by the previously described traits. We determined 95% confidence intervals and significance using an ultimate cluster model for variance estimation. All analysis was done in Matlab R2022b (MathWorks, Natick, MA) as well as SAS v 9.4 (SAS Institute, Inc, Cary, NC).

## RESULTS

### General Characteristics, Demographics and Diagnostics of Patients Presenting with Vaginal Bleeding

From 2011–2019, the NHAMCS published data representative of 1,241,074,996 ED encounters, of which 14,620,933 were for adult visits related to vaginal bleeding (1.3% [95% CI, 1.2–1.4%] of all encounters, and 2.7% [95% CI, 2.4–2.9%] of all adult female encounters). Approximately half of these encounters (53.0% [95% CI, 49.4–56.7%]) were identified as encounters of pregnant patients, and over 90% (90.9% [95% CI, 89.2–92.7%]) were for patients in the pre-menopausal age cohort (between 18–44 years of age). Among all patients, there was a lower prevalence of White patients presenting with this complaint compared to White patients presenting with any chief complaint (45.6% [95% CI, 41.9–49.4] vs 60.3% [95% CI, 57.7–62.8%]), with a reciprocal higher prevalence of Hispanic (21.1% [95% CI, 17.7–24.5%] vs 13.2% [95% CI, 11.7–14.8%,]) and Black patients (30.2% [95% CI, 25.9–34.5] vs 23.5% [95% CI, 20.9–26.2%]) (Table 1).

In general, pregnant patients were significantly younger (27.2 years [95% CI, 26.7–27.8)] compared to non-pregnant patients (33.4 years [95% CI, 32.3–34.5]). There were no significant differences or trends appreciated in ESI scores by pregnancy status, age cohort, or race/ethnicity. There was a general trend without significance toward shorter wait times for pregnant (39.6 minutes [95% CI, 34.0–45.2] vs 41.3 [95% CI, 35.4–47.2] in non-pregnant), post-menopausal age cohort (28.7 minutes [95% CI, 13.9–43.5] vs 44.7 minutes [95% CI, 30.7–58.7] in peri- and 40.5 minutes [95% CI, 25.9–45.1, 95] in pre-menopausal), and White patients (35.8 minutes [95% CI, 30.4–41.2] vs 45.1 minutes [95% CI, 35.8–54.3] in Black and 44.1 minutes [95% CI, 34.4–53.8] in Hispanic patients) (Table 2). Overall, 88.1% (95% CI, 86.1–90.0) of patients were discharged home directly from the ED and, thus, met criteria for low-severity outcome; 10.3% (95% CI, 8.5–12.1) met criteria for moderate-severity outcome; and 1.6% (95% CI, 1.0–2.2) met criteria for having a high-severity outcome (Table 2).

Encounters classified as resulting in high-severity outcomes, as compared to low-severity outcomes, were found to have significantly shorter wait times (23.4 minutes [95% CI, 17.1–29.8] vs 41.7 [95% CI, 37.1–46.4[]), lower diastolic blood pressures (71.8 mm Hg [95% CI, 8.5–75.1] vs 76.4 [95% CI, 75.6–77.3]), higher heart rates (92.6 beats per minute [95% CI, 87.5–97.6] vs 85.4 [95% CI, 84.5–86.4]), and higher shock indices (0.75 [95% CI, 0.72–0.78] vs 0.68 [95% CI, 0.67–0.69]) (Table 3). There was no difference in median ESI score or use of imaging diagnostics by ultimate outcome severity.

### Stratifications by Subgroup

Patients were further stratified by estimated pregnancy status, age cohort, and race/ethnicity. This was done to identify how presenting demographics might be associated with clinical characteristics such as vitals, diagnostic pathways such as imaging and pelvic exams and, most importantly, ultimate outcomes and dispositions (Tables 4 and 5).

Stratification by pregnancy status demonstrated a small but statistically significant difference in SI (0.71 [95% CI, 0.69–0.72, 95% CI] in pregnant patients vs 0.67 [95% CI, 0.66–0.68] in non-pregnant patients), a significantly lower rate of CT imaging (0.9% [95% CI, 0–2.1%] vs 7.1% [95% CI, 5.0–9.2%,), and a significantly higher rate of ultrasound (64.4% [95% CI, 60.0–68.7%] vs 26.7% [95% CI, 22.6–30.9%]). No significant variations in pelvic exams or outcome disposition by pregnancy status were found (Table 4).

Patients in the post-menopausal age cohort were significantly more hypertensive than patients in the pre-menopausal cohort (147.1 mm Hg [95% CI, 140.2–154.0] vs 126.3 mm Hg [95% CI, 125.2–127.5]), with a resulting decrease in SI (0.58 [95% CI, 0.53–0.62] vs 0.70 [95% CI, 0.69–0.71]). Patients in the pre-menopausal cohort were significantly more likely to undergo ultrasound imaging (48.3% [95% CI, 44.8–51.8%] vs 32.0% [95% CI, 21.4–42.6%] in peri- and 28.0% [95% CI, 14.6–41.5%] in post-menopausal age cohorts). Age cohort was not associated with differences in high-severity outcome (Table 4).

Stratification by race/ethnicity produced no clinically meaningful differences in triage vitals, diagnostic testing, or ultimate outcome severity. Hispanic patients were significantly more likely to be pregnant (65.4% [95% CI, 58.8–72.0%]) as compared to White (50.8% [95% CI, 45.7–55.9%]) or Black patients (47.6% [95% CI, 41.2–54.0%) and to undergo ultrasound imaging (58.7% [95% CI, 51.6–65.8%]) than any other group (44.8% [95% CI, 40.2–49.5%] in White and 41.4% [95% CI, 35.7–47.0%] in Black patients) (Table 5).

## DISCUSSION

This study represents the largest analysis to date of all patients presenting to US EDs with a chief complaint related to vaginal bleeding. It is the first to estimate ED utilization by initial chief complaint of vaginal bleeding and to evaluate factors associated with poor outcomes from initial presenting characteristics. As restrictions on safe pregnancy terminations increase across the country, understanding who presents to EDs with this chief complaint and which characteristics are associated with poor outcomes is more important now than ever.

We found that most patients presenting to EDs for vaginal bleeding were in the pre-menopausal age cohort (18–44 years of age). Despite representing approximately half of the total ED population, this cohort makes up > 90% of the population presenting with vaginal bleeding. Moreover, within this age group, most of these patients were identified as pregnant. This confirms what most emergency physicians likely expect from experience—that the largest group of patients presenting with vaginal bleeding in the emergent setting are young and pregnant.

Worrisomely, we found discrepancies in presentation for vaginal bleeding by race/ethnicity. We found a significantly lower proportion of White patients presenting with vaginal bleeding as a chief complaint compared to the general population representation, a significantly higher proportion of Hispanic patients, and a trend without significance toward a higher proportion of Black patients. The initial inclination might be to postulate that Black and Hispanic patients use the ED more for primary care needs due to discrepancies in primary care access; however, we found no difference in outcome severity by race/ethnicity, suggesting these groups were not accessing emergency care for lower acuity needs. Additionally, there were no significant differences in the presenting vitals, assigned ESI, or wait times of these groups to suggest one cohort might be more or less sick than another. The factors influencing a patient’s decision to seek and the need for emergent care, particularly in the setting of racial differences, warrants further investigation.

When examining what presenting characteristics were associated with ultimate outcome severity, we found statistically significant higher heart rates and SI in those who were ultimately classified as having high-severity outcomes. This finding fits with clinical expectations, as the primary concern in severe vaginal bleeding is hemorrhagic shock, which would first present as elevations in heart rate and SI. Despite no significant difference in triage scores, there was a significant trend in patients being seen faster who were later classified as having high-severity outcomes, on average nearly twice as fast as those ultimately classified as having low-severity outcomes. This is a hopeful finding, as it suggests triage staff are appropriately taking clinical context into account when assigning rooms despite triage scores being insensitive to these differences.

In evaluating what diagnostic tests are performed for patients with vaginal bleeding, we found the most common imaging modality across every subgroup was ultrasound, with some notable patterns. As expected, pregnant patients were more than twice as likely to undergo ultrasound imaging than their non-pregnant counterparts. Computed tomography was a relatively rare imaging modality across cohorts, with a trend without significance toward higher use in post-menopausal patients. Hispanic patients were marginally more likely to undergo ultrasound; however, this may have been attributable to statistically higher rate of pregnancy in Hispanic patients presenting with vaginal bleeding.

We found that less than a quarter of the patients presenting with vaginal bleeding received a documented pelvic exam, and that this rate remained approximately the same without significant differences regardless of stratification of patients by pregnancy status, age cohort, race/ethnicity, or outcome severity. This warrants further discussion, as whether to perform a pelvic exam is likely influenced by many factors. The ACOG acknowledges that routine screening pelvic exams are likely not evidence based^17^ but recommends that they be performed when indicated by medical history or symptoms, citing abnormal bleeding as one reason why an exam should be performed.^18^ The popular clinical guide UpToDate recommends pelvic exams in any examination of a patient with vaginal bleeding, with some exception given to bleeding later in pregnancy.^19^ The American College of Emergency Physicians currently has no clinical recommendation on this issue. In our study, the rate of pelvic exams remained stable across patient subgroups. This would suggest that the decision to perform a pelvic exam in these patients was driven less by patient characteristics and more by individual practice patterns of clinicians.

Finally, in terms of ultimate disposition, we found that most patients presenting with vaginal bleeding were discharged home, with only a small percentage meeting criteria for high-severity outcomes. There were no significant differences in these rates across any of the subgroups, a somewhat unexpected result since this complaint represents likely vastly different physiologic processes across pregnancy status and age cohorts. A clinically meaningful takeaway from these numbers would be that approximately 10% of these patients are admitted or held in observation, with 1–2% requiring more emergent attention.

## LIMITATIONS

There are several limitations to this study that warrant discussion. Specifically, as a cross-sectional study, only information regarding one hospital encounter was captured. Patients were not followed over the longitudinal course of their illness: future adverse events warranting additional ED visits or hospitalizations were not captured. Additionally, some data elements not specifically collected must be inferred, such as pregnancy status and estimated menopausal status.

Although the use of a national sample strengthens the generalizability of our findings, it is important to note that our results may underestimate the prevalence of vaginal bleeding in the emergent setting. Due to an array of societal and cultural elements, some patients may still feel the need to conceal initial concerns regarding vaginal bleeding. Since this study design identifies cases by initial chief complaint, it may miss encounters later identified to have a concern for vaginal bleeding.

Finally, the structure and compilation of the dataset itself is a limitation. The NHAMCS dataset comes in the form of a raw undelimited text file, with variable structure year to year, requiring manual extraction and parsing. Combining multiple years of NHAMCS data introduces further limitations, as variable data collection limits which variables can be reliably used. As a down-sampled representative survey sample, rare events can be missed or inappropriately represented by chance, even despite following NHAMCS best practice guidelines. For instance, there were no documented instances of mortality in this cohort of patients presenting with vaginal bleeding. Finally, as in any dataset, there were missing data, and some data fields were likely more prone to missing data than others. Procedures, such as pelvic exams, likely underestimate true incidence as they may well be performed without specific documentation capturing that event.

## CONCLUSION

This study affirms what many emergency physicians experience anecdotally: that most patients with vaginal bleeding are young, pregnant, and discharged home. Patients who have severe outcomes are seen faster, despite no differences in their initial triage scores. It raises alarming questions regarding the disproportionate representation of patients from minority groups, without a reciprocal difference in admission or severity outcomes. Future research is needed to examine disparities in presentation and factors associated with poor outcomes, in particular, racial/ethnic differences in access to primary obstetric care and its impact on adverse outcomes. Finally, this study identifies that although most patients undergo ultrasound imaging, only about one quarter of patients presenting with vaginal bleeding undergo a pelvic examination, with no significant differences by subgroup. These findings suggest that guidance on which patients presenting to the ED setting with vaginal bleeding would benefit from a pelvic exam may be warranted.

## Supplementary Information



## Figures and Tables

**Table 1:** Baseline characteristics of patients presenting to the emergency department with vaginal bleeding.

	Weighted frequency	Percentage of vaginal bleeding visits (95% CI)
All patients with vaginal bleeding	14,620,933	100
Pregnancy status		
Pregnant	7,757,173	53.0 (49.4, 56.7)
Not pregnant	6,863,760	47.0 (43.3, 50.6)
Age cohort		
Pre-menopausal (18–44)	13,296,252	90.9 (89.2, 92.7)
Peri-menopausal (45–55)	915,046	6.3 (4.9, 7.7)
Post-menopausal (≥ 56 years of age)	409,635	2.8 (1.9, 3.7)
Race/ethnicity		
White (non-Hispanic)	6,676,750	45.6 (41.9, 49.4)
Black (non-Hispanic)	4,413,079	30.2 (25.9, 34.5)
Hispanic	3,081,906	21.1 (17.7, 24.5)
Other (non-Hispanic)	449,198	3.1 (1.9, 4.3)

**Table 2:** Emergency Severity Index scores, wait times, and outcome severity of emergency department patients presenting with vaginal bleeding.

	Age, Mean (95% CI), Years	ESI, median (IQR)	ESI, mode	Wait time, mean (95% CI), minutes	Percentage Outcome Severity (95% CI)*		

					Low	Moderate	High
All patients with vaginal bleeding	30.1 (29.4, 30.8)	2.6 (2.2, 2.9)	3	40.4 (35.9, 44.9)	88.1 (86.1, 90.0)	10.3 (8.5, 12.1)	1.6 (1.0, 2.2)
Pregnancy status							
Pregnant	**27.2 (26.7, 27.8)**	2.6 (2.2, 2.9)	3	39.6 (34.0, 45.2)	89.8 (87.8, 91.8)	8.4 (6.6, 10.2)	1.8 (1.0, 2.6)
Not pregnant	**33.4 (32.3, 34.5)**	2.6 (2.3, 2.9)	3	41.3 (35.4, 47.2)	86.1 (82.8, 89.4)	12.5 (9.3, 15.7)	1.4 (0.7, 2.2)
Age cohort							
Pre-menopausal (18–44 years)	27.7 (27.2, 28.1)	2.6 (2.2, 2.9)	3	40.5 (25.9, 45.1)	88.8 (86.8, 90.7)	9.6 (7.8, 11.3)	1.7 (1.0, 2.3)
Peri-menopausal (45–55 years)	49.1 (48.4, 49.7)	2.5 (2.2, 2.8)	3	44.7 (30.7, 58.7)	78.6 (69.4, 89.9)	20.0 (10.8, 29.3)	1.3 (0.0, 2.8)
Post-menopausal (≥ 56 years)	67.7 (65.3, 70.2)	2.6 (2.2, 2.9)	3	28.7 (13.9, 43.5)	85.7 (86.8, 90.7)	12.5 (3.4, 21.6)	1.8 (0.0, 4.6)
Race/Ethnicity							
White (non-Hispanic)	30.5 (29.4, 31.5)	2.6 (2.2, 2.9)	3	35.8 (30.4, 41.2)	88.6 (85.8, 91.5)	9.1 (6.6, 11.5)	2.3 (1.2, 3.4)
Black (non-Hispanic)	28.9 (27.8, 30.1)	2.6 (2.3, 2.9)	3	45.1 (35.8, 54.3)	87.5 (84.4, 90.6)	11.5 (8.5, 14.5)	0.9 (0.3, 1.6)
Hispanic	30.6 (29.4, 31.7)	2.6 (2.2, 2.9)	3	44.1 (34.4, 53.8)	87.8 (84.3, 91.3)	10.9 (7.5, 14.2)	1.3 (0.3, 2.3)
Other (non-Hispanic)	34.0 (30.9, 37.0)	2.4 (2.1, 2.7)	3	38.4 (23.2, 53.6)	86.0 (76.8, 95.2)	13.2 (3.9, 22.4)	0.8 (0.0, 2.0)

* High-severity outcome was defined as any patient who was dead on arrival, died in the ED or during that hospitalization, was admitted to the intensive care or stepdown units, to the cardiac catheterization lab or the operating room, or transferred to a non-psychiatric hospital. Moderate severity was defined as any patient admitted to floor level care, held in observation, or transferred to a psychiatric hospital. Low-severity outcome was defined as any patient discharged home.

Bolded values represent significant results with p<0.05 (for this and all table legends)

*ED*, emergency department; *ESI*, emergency severity index.

**Table 3:** Demographics and diagnostics of emergency department patients presenting with vaginal bleeding, by ultimate outcome severity.

	Outcome severity*		

	Low	Moderate	High
Weighted frequency	12,875,347	1,507,975	237,610
Percentage (95% CI)	88.1 (86.1, 90.0)	10.3 (8.5, 12.1)	1.6 (1.0, 2.2)
ESI, median (IQR)	2.6 (2.2, 2.9)	2.6 (2.3, 3.0)	2.5 (2.1, 2.8)
ESI, mode	3	3	3
Wait time, (minutes) mean (95% CI)	**41.7 (37.1, 46.4)**	31.8 (20.4, 43.2)	**23.4 (17.1, 29.8)**
Vitals			
Heart rate, mean (95% CI), beats/min	**85.4 (84.5, 86.4)**	88.7 (86.0, 91.4)	**92.6 (87.5, 97.6)**
Respiratory rate, mean (95% CI), breaths/min	17.7 (17.5, 17.9)	18.2 (17.4, 19)	17.9 (17.2, 18.5)
Systolic blood pressure, mean (95% CI), mm Hg	128 (126.8, 129.2)	124.6 (121.3, 127.9)	124.9 (120.9, 128.9)
Diastolic blood pressure, mean (95% CI), mm Hg	**76.4 (75.6, 77.3)**	73.8 (71.9, 75.6)	71.8 (68.5, 75.1)
Pulse oximetry, mean (95% CI), percentage oxygen saturation	98.4 (98.2, 98.6)	98.6 (98.2, 99)	98.1 (97.4, 98.8)
Temperature, mean (95% CI), degrees Fahrenheit	98.2 (98.2, 98.3)	98.2 (98.1, 98.4)	98.3 (98.1, 98.5)
Shock Index, mean (95% CI)	**0.68 (0.67, 0.69)**	0.74 (0.7, 0.77)	**0.75 (0.72, 0.78)**
Diagnostic exams			
CT abdomen/pelvis, percentage (95% CI)	3.5 (2.4, 4.5)	2.5 (0.4, 4.6)	5.7 (1.5, 9.9)
Ultrasound, percentage (95% CI)	47.7 (43.9, 51.5)	36.2 (28.3, 44.1)	57.0 (38.6, 75.3)
Pelvic exam, percentage (95% CI)	24.4 (20.7, 28.0)	23.8 (14.7, 33.0)	26.3 (8.7, 44.0)

* High severity outcome was defined as any patient who was dead on arrival, died in the ED or during that hospitalization, was admitted to the intensive care or stepdown units, to the cardiac catheterization lab or the operating room, or transferred to a non-psychiatric hospital. Moderate severity was defined as any patient admitted to floor level care, held in observation, or transferred to a psychiatric hospital. Low severity outcome was defined as any patient discharged home.

*CT*, computed tomography*; ESI*, Emergency Severity Index.; *mm Hg*, millimeters of mercury.

**Table 4:** Vital signs, diagnostics, and outcome severity of emergency department patients presenting with vaginal bleeding, by pregnancy status and age cohort.

	Pregnancy status		Age cohort		
	
	Pregnant	Not pregnant	Pre-menopausal (18–44 years)	Peri-menopausal (45–55 years)	Post-menopausal (≥ 56 years)
Vitals					
Heart rate, mean (95% CI), beats/min	86.2 (84.9, 87.4)	85.6 (84.5, 86.7)	85.9 (85, 86.8)	87.1 (83.7, 90.4)	83.1 (77.2, 89)
Respiratory rate, mean (95% CI), breaths/min	17.7 (17.5, 17.8)	17.9 (17.6, 18.2)	17.7 (17.6, 17.9)	18 (17.6, 18.3)	18.2 (17.8, 18.6)
Systolic blood pressure, mean (95% CI), mm Hg	124.8 (123.4, 126.2)	130.7 (129.1, 132.4)	**126.3 (125.2, 127.5)**	137.1 (133.2, 141)	147.1 (140.2, 154)
Diastolic blood pressure, mean (95% CI), mm Hg	74 (73, 75.1)	78.4 (77.4, 79.3)	75.7 (74.8, 76.5)	80.6 (78.5, 82.7)	78.8 (75.1, 82.5)
Pulse oximetry, mean (95% CI), percentage oxygen saturation	98.6 (98.3, 98.8)	98.3 (98, 98.5)	98.5 (98.3, 98.7)	98.2 (97.4, 99)	96.7 (94.5, 98.9)
Temperature, mean (95% CI), °F	98.3 (98.2, 98.3)	98.2 (98.1, 98.2)	98.2 (98.2, 98.3)	98.2 (98.1, 98.4)	98.1 (97.8, 98.3)
Shock Index, mean (95% CI)	**0.71 (0.69, 0.72)**	**0.67 (0.66, 0.68)**	**0.70 (0.69, 0.71**)	0.65 (0.62, 0.69)	**0.58 (0.53, 0.62)**
Diagnostic exams					
CT abdomen/pelvis, percentage (95% CI)	**0.8 (0, 1.8)**	**6.3 (4.5, 8.2)**	2.8 (1.8, 3.7)	9.3 (3.3, 15.3)	10 (1.8, 18.2)
Ultrasound, percentage (95% CI)	64.4 (60, 68.7)	26.7 (22.6, 30.9)	48.3 (44.8, 51.8)	32 (21.4, 42.6)	28 (14.6, 41.5)
Pelvic exam, percentage (95% CI)	23.7 (19.1, 28.3)	24.9 (20.5, 29.3)	23.7 (20.3, 27.2)	29.3 (18.5, 40.2)	33.9 (21.7, 46.1)
Outcome severity*					
Low, percentage (95% CI)	86.1 (82.8, 89.3)	89.8 (87.8, 91.8)	88.8 (86.8, 90.7)	78.6 (70.3, 87)	85.7 (78.5, 92.8)
Moderate, percentage (95% CI)	12.5 (9.3, 15.7)	8.4 (6.6, 10.2)	9.6 (7.8, 11.3)	20 (11.7, 28.4)	12.5 (6, 19)
High, percentage (95% CI)	1.4 (0.7, 2.2)	1.8 (1, 2.6)	1.7 (1, 2.3)	1.3 (0, 2.8)	1.8 (0, 4.5)

* High-severity outcome was defined as any patient who was dead on arrival, died in the ED or during that hospitalization, was admitted to the intensive care or stepdown units, to the cardiac catheterization lab or the operating room, or transferred to a non-psychiatric hospital. Moderate severity was defined as any patient admitted to floor level care, held in observation, or transferred to a psychiatric hospital. Low-severity outcome was defined as any patient discharged home.

*CT*, computed tomography; *mm Hg*, millimeters of mercury.

**Table 5:** Vital signs, diagnostics, and outcome severity of emergency department patients presenting with vaginal bleeding, by race/ethnicity.

	White (non-Hispanic)	Black (non-Hispanic)	Hispanic	Other (non-Hispanic)
Vitals				
Heart rate, mean (95% CI), beats/min	87.0 (85.4, 88.6)	85.2 (84.1, 86.3)	84.4 (82.9, 85.9)	87.3 (82.9, 91.7)
Respiratory rate, mean (95% CI), breaths/min	17.8 (17.5, 18.1)	17.8 (17.5, 18.1)	17.7 (17.4, 18.1)	17.5 (16.9, 18.1)
Systolic blood pressure, mean (95% CI), mm Hg	128.7 (126.9, 130.4)	128.4 (126.6, 130.1)	124.5 (122.4, 126.6)	125.7 (121.3, 130.2)
Diastolic blood pressure, mean (95% CI), mm Hg	76.8 (75.5, 78.0)	76.3 (75, 77.6)	74.7 (73.4, 76)	73.4 (71.3, 75.4)
Pulse oximetry, mean (95% CI), percent oxygen saturation	98.2 (97.9, 98.5)	98.7 (98.4, 99.0)	98.5 (98.2, 98.8)	98.7 (98.2, 99.1)
Temperature, mean (95% CI), °Fahrenheit	98.2 (98.2, 98.3)	98.3 (98.2, 98.3)	98.2 (98.1, 98.2)	98.2 (98, 98.4)
Shock Index, mean (95% CI)	0.69 (0.68, 0.71)	0.68 (0.67, 0.69)	0.69 (0.68, 0.71)	0.71 (0.68, 0.74)
Diagnostic exams				
CT abdomen/pelvis, percentage (95% CI)	3.4 (2.1, 4.7)	3.1 (0.9, 5.2)	4 (1.5, 6.4)	2.1 (0, 5.6)
Ultrasound, percentage (95% CI)	**44.8 (40.2, 49.5)**	**41.4 (35.7, 47)**	**58.7 (51.6, 65.8)**	44.2 (32.4, 56.1)
Pelvic exam, percentage (95% CI)	25.3 (20.9, 29.6)	24 (17.9, 30)	23.3 (17.1, 29.4)	21.5 (13, 30.1)
Outcome severity*				
Low, percentage (95% CI)	88.6 (85.8, 91.5)	87.5 (84.5, 90.6)	87.8 (84.4, 91.2)	86 (77, 95)
Moderate, percentage (95% CI)	9.1 (6.6, 11.5)	11.5 (8.5, 14.5)	10.9 (7.6, 14.1)	13.2 (4, 22.3)
High, percentage (95% CI)	2.3 (1.2, 3.4)	0.9 (0.3, 1.6)	1.3 (0.3, 2.4)	0.8 (0, 2)

* High-severity outcome was defined as any patient who was dead on arrival, died in the ED or during that hospitalization, was admitted to the intensive care or stepdown units, to the cardiac catheterization lab or the operating room, or transferred to a non-psychiatric hospital. Moderate severity was defined as any patient admitted to floor level care, held in observation, or transferred to a psychiatric hospital. Low-severity outcome was defined as any patient discharged home.

*CT*, computed tomography; *mm Hg*, millimeters of mercury.
