# Supplementary material for: Epidemiology and Outcomes of Patients Presenting to United States Emergency Departments with Vaginal Bleeding
Source: West J Emerg Med. 2026 Feb 3;27(2):321–9. doi: 10.5811/westjem.49015 (PMC13016043; doi:10.5811/westjem.49015)
Supplement: Supplementary file 1 [file wjem-27-321-s001.docx]

**APPENDIX 1**

**NHAMCS Reason for Visit Codes related to vaginal bleeding:**

Included

1735.1 Irregularity of menstrual interval, frequent

1740.1 Excessively heavy flow

1740.3 Abnormal material including clots

1755.0 Uterine and vaginal bleeding

1755.1 Intermenstrual bleeding

1755.2 Postmenopausal bleeding

1755.3 Postcoital bleeding

1790.2 Bleeding during pregnancy

Notable codes not included

1740.0 Irregularity of flow

1760.0 Vaginal discharge, including bloody, brown, white, excessive

1791.0 Postpartum problems, including bleeding, pain

Notably, three codes were excluded from analysis because they were not specific to increased frequency or quantity of vaginal bleeding. These codes were 1740.0 (irregularity of flow), 1760.0 (vaginal discharge, including bloody, brown, white, excessive) and 1791.0 (postpartum problems, including bleeding, pain). In total, these three codes excluded 809 unique encounters, representing approximately 4.7 million ED visits. Since patients could have multiple reasons for their visit listed, if both an inclusion code, as well as one of the three excluded codes above were listed, they were included.

**NHAMCS Reason for Visit Codes related to pregnancy:**

Included

1790.0 Problems and other related to pregnancy

1790.1 Pain during pregnancy

1790.2 Bleeding during pregnancy

1790.3 Symptoms related to onset of labor

2735.0 Diagnosed complications of pregnancy

3515.0 Counseling and examinations for pregnancy interruption

3520.0 Abortion to be performed

Notable codes not included

3200.0 Pregnancy, unconfirmed

3205.0 Prenatal exam

3500.0 Family planning (includes unwanted pregnancy, contraceptive NOS, but

also includes others not relevant)

**ICD Codes related to pregnancy:**

ICD 10: Pregnancy, childbirth and the puerperium (O00-O9A)

ICD 9:  Complications of pregnancy, childbirth and the puerperium (630-679)
